# Safety and treatment patterns of multikinase inhibitors in patients with metastatic renal cell carcinoma at a tertiary oncology center in Italy

**DOI:** 10.1186/1471-2407-11-105

**Published:** 2011-03-24

**Authors:** Camillo Porta, Chiara Paglino, Ilaria Imarisio, Cinzia Canipari, Kristina Chen, Maureen Neary, Mei Sheng Duh

**Affiliations:** 1 Medical Oncology, I.R.C.C.S. San Matteo University Hospital Foundation, Piazzale C. Golgi 19, 27100 Pavia, Italy; 2 Analysis Group, Inc., Boston, Massachusetts, USA; 3 GlaxoSmithKline, Collegeville, Pennsylvania, USA

## Abstract

**Background:**

Multikinase inhibitors (MKIs) sunitinib and sorafenib have become a standard of care for metastatic renal cell carcinoma (mRCC). This study assessed safety and treatment patterns for these agents in a real-world clinical practice setting in Italy.

**Methods:**

A retrospective medical record review was performed at a tertiary oncology center in Italy. The study included MKI-naïve non-trial patients ≥18 years old, with a histological diagnosis of mRCC, and who received sunitinib or sorafenib as first MKI during 9/2005-7/2008. Data were collected on adverse events (AEs), treatment modifications (discontinuations, interruptions, dose changes), and reasons for these modifications.

**Results:**

145 patients were included; 85 received sunitinib and 60 received sorafenib as first-line MKI. Median treatment duration was 6.6 (sunitinib) and 5.8 (sorafenib) months. 97.6% and 70.0% of patients receiving sunitinib and sorafenib, respectively, experienced ≥1 AE; 27.1% and 31.7% had ≥1 grade 3/4 AE. The most common any grade AE for sunitinib was fatigue/asthenia (81.2%), followed by mucositis/stomatitis (58.8%) and decreased taste sensation (42.4%), while for sorafenib this was fatigue/asthenia (43.3%) followed by hand-foot syndrome (38.3%) and diarrhea (31.7%). Treatment discontinuation, interruption, and dose reduction due to AEs occurred in 11.8%, 23.5%, and 30.6%, respectively, of patients receiving sunitinib, and 5.0%, 23.3%, and 36.7%, respectively, of patients receiving sorafenib.

**Conclusions:**

In this retrospective study, most patients experienced ≥1 AE during first-line MKI treatment. AEs were reported frequently and resulted in treatment modifications in 40% of patients receiving sunitinib and 45% of patients receiving sorafenib. These results suggest a need for additional effective and more tolerable treatments for mRCC.

## Background

Treatment options for metastatic renal cell carcinoma (mRCC) have grown to include anti-angiogenic agents, which inhibit the vascular endothelial growth factor (VEGF) pathway and disrupt tumor growth. Sunitinib and sorafenib are oral multikinase inhibitors (MKIs) that have received approval for treatment of RCC in Europe and the U.S. and have become a standard of care in mRCC. Both agents have demonstrated efficacy in tumor shrinkage and prolonged progression-free survival (PFS) of patients with advanced or metastatic RCC within randomized clinical trials [1-4].

Clinical trials showed that sunitinib and sorafenib are commonly associated with certain adverse events, including fatigue, diarrhea, hypertension and dermatologic toxicities (rash and hand-foot skin reaction) [1-4]. Other adverse events reported in clinical trials among patients treated with sunitinib included nausea, stomatitis, vomiting, and mucosal inflammation [1,3]. Patients treated with sorafenib also reported alopecia, nausea, and anorexia [2,4].

Clinical trials may not be representative of real-life clinical practice due to strict and homogeneous patient selection criteria, as patients in clinical trials generally have fewer comorbidities than those seen in oncology practice so that the effect of the active treatment can be better isolated [5]. Thus, observational studies in patients treated at clinical settings are needed in addition to clinical trials to further examine the safety profiles of sunitinib and sorafenib. Expanded-access trials, which enrolled primarily patients who were not eligible to participate in clinical trials due to exclusion criteria, have been conducted to examine the efficacy and safety of sunitinib and sorafenib. In an expanded-access trial for sunitinib that enrolled 4,564 patients, the most common treatment-related all-grade adverse events were diarrhea (44%) and fatigue (37%), and the most common grade 3/4 adverse event was fatigue (8%) [6]. Reasons for discontinuation included lack of efficacy (27%) and adverse events (8%). In an expanded-access trial for sorafenib that enrolled 2,502 patients [7], the most common drug-related adverse events were rash (26%) and hand-foot syndrome (23%), and the most common grade 3/4 adverse event was hand-foot syndrome (8%). Adverse events resulted in treatment discontinuation in 20% of patients.

Based on data from everyday clinical practice adverse events among patients taking sunitinib or sorafenib may be higher than those observed and reported from clinical trials. For example, thyroid dysfunctions, which have now been identified as one of the frequent tyrosine kinase-related adverse events, were not reported as such in the pivotal clinical trial of sunitinib [1,8]. Furthermore, toxicities associated with both sunitinib and sorafenib appeared to be higher in the two global expanded access programs (EAPs) and in reports from single centers compared to that in the clinical trials [6,7,9]. The primary objective of this study was to examine the safety profiles of sunitinib and sorafenib and the frequency of treatment modifications, including treatment discontinuation, treatment interruptions and dose changes in a real-world setting at a tertiary oncology center.

## Methods

### Study Design and Data Source

In this retrospective, observational study, medical records of eligible patients treated at IRCCS San Matteo University Hospital, Pavia, Italy, were reviewed. Data extracted from the medical records included but were not limited to: date of initial RCC diagnosis, demographic variables, comorbidities, prior pharmacological or radiological treatments, metastatic site(s), baseline Eastern Cooperative Oncology Group (ECOG) performance status (PS) score, drug-related adverse event data, laboratory data, and radiologic test results. Other treatment-related data collected included dates of treatment initiation and discontinuation, initial dosing, dates and reasons of treatment interruptions and treatment changes, dosing modifications and follow-up tumor assessments.

The observation period for each patient extended from the initiation of the first MKI therapy to the earliest of death, loss to follow up, or end of the study period. Patients who switched to another MKI during the observation period continued to be followed in the study, but their observation periods were analyzed separately as first-line and second-line treatment. Data on patients who received the first MKI therapy between September 2005 and July 2008 were included. This study was approved by the San Matteo University Hospital's Ethics Committee.

### Study Population

Patients aged 18 years or older with histologically or cytologically confirmed mRCC who were MKI-naïve prior to the first dose of sunitinib or sorafenib were eligible to be included in the study. Patients could have received prior immunotherapy and/or chemotherapy. Eligible patients were required to have received at least one dose of sunitinib and/or sorafenib after January 1, 2005. Patients who were enrolled in a RCC clinical trial at any time during this study or had previously enrolled in a RCC clinical trial within 6 months prior to the initiation of MKI therapy were excluded from the study, but patients who participated in EAPs were permitted. Patients who had less than 3 months of follow-up data were excluded to ensure an adequate amount of information.

### Outcome Definitions

#### Safety

Safety outcomes included the numbers and proportions of patients who experienced specific adverse events, of any grade and of grade 3 or higher. The study investigators retrospectively assessed toxic events and assigned grade levels using the National Cancer Institute's Common Terminology Criteria for Adverse Events (CTCAE), version 3.0, at chart abstraction because grade levels of adverse events are not regularly recorded in medical charts in clinical settings [10].

#### Treatment Patterns

Outcomes of treatment patterns included the numbers and proportions of patients who had a treatment discontinuation, interruption, or dose change during first-line MKI treatment, and those who switched to a second-line MKI treatment. The type, date, and the reasons for treatment modification were abstracted from patient medical charts. A treatment interruption occurred if therapy was held and then later resumed. If treatment was stopped and never resumed, the patient was classified as discontinuing therapy.

### Statistical Analyses

Descriptive statistics were used to describe patient baseline characteristics, adverse events, and treatment patterns. Means, median, and ranges were used to describe continuous variables; frequencies and percentages were reported for categorical variables. Median treatment duration was assessed using the Kaplan-Meier survival method. All data analyses were conducted using SAS software, version 9.1.

## Results

### Patient Characteristics

Table 1 shows the baseline demographic and clinical characteristics of the study patients. A total of 145 patients were included in this study. Among them, 85 patients received sunitinib and 60 patients received sorafenib as first-line MKI. All 85 patients receiving sunitinib initiated treatment at the recommended dose of 50 mg once daily, 4 weeks on, followed by 2 weeks off (QD, 4/2). 98.3% of patients receiving sorafenib initiated treatment at the recommended dose of 400 mg twice daily.

**Table 1:** Baseline Characteristics among Patients Receiving First-Line Multikinase Inhibitor Treatment

	Sunitinib	Sorafenib
	(N = 85)	(N = 60)
**Initial Dose, N (%)**	50 mg QD 85 (100.0)	400 mg BID 59 (98.3) 400 mg QD 1 (1.7)

**Age at initiation, years**		

Median (range)	62.4 (35.1-81.9)	66.0 (37.9-77.7)

Mean (std.)	60.1 (10.4)	63.2 (9.7)

**Male, N (%)**	74 (87.1)	53 (88.3)

**ECOG Performance Status, N (%)**		

0	69 (81.2)	35 (58.3)

1	14 (16.5)	13 (21.7)

2	0 (0.0)	1 (1.7)

NA	2 (2.4)	11 (18.3)

**Number of metastatic sites**		

1	8 (9.4)	9 (15.0)

2	30 (35.3)	9 (15.0)

3	25 (29.4)	21 (35.0)

>3	22 (25.9)	21 (35.0)

**Metastatic sites, N (%)**		

Bone	29 (34.1)	18 (30.0)

Brain	14 (16.5)	10 (16.7)

Liver	18 (21.2)	23 (38.3)

Lung	59 (69.4)	46 (76.7)

Lymph nodes	52 (61.2)	36 (60.0)

**Previous therapy, N (%)**		

Radiotherapy	30 (35.3)	21 (35.0)

Nephrectomy	84 (98.8)	58 (96.7)

Immunotherapy	68 (80.0)	49 (81.7)

Chemotherapy	39 (45.9)	28 (46.7)

NA = not available

Most patients in both groups had a baseline ECOG PS score of 0 (81.2% for sunitinib and 58.3% for sorafenib); only 1 patient in the sorafenib group had an ECOG PS score of 2. More than half of all patients had 3 or more metastatic sites at the baseline. The most prevalent metastatic sites were lung and lymph nodes (60.0%-76.7%); 16.5% of patients receiving sunitinib and 16.7% of patients receiving sorafenib had brain metastases at baseline. Almost all patients had a history of nephrectomy and immunotherapy (Table 1).

### Safety

Table 2 presents the frequencies and rates of all-grade and grade 3/4 adverse events observed in the study population, as reported in patients' medical charts. Among patients receiving sunitinib and sorafenib, 97.6% and 70.0%, respectively, experienced at least one adverse event. The most common all-grade adverse event for both MKIs was fatigue or asthenia, observed in 81.2% of patients receiving sunitinib and 43.3% of patients receiving sorafenib. In patients receiving sunitinib, other frequently reported all-grade adverse events included mucositis or stomatitis (58.8%) and decreased taste sensation (42.4%). In patients receiving sorafenib, hand-foot syndrome was the second most frequently reported all-grade adverse event (38.3%), followed by diarrhea (31.7%). Fatigue or asthenia was also the most common grade 3/4 adverse events for both agents, reported in 9.4% of patients receiving sunitinib and 10.0% of patients receiving sorafenib. Other frequently reported grade 3/4 adverse events included anorexia and vomiting (5.9% each) and hypertension, nausea, and abdominal pain (3.5% each) in patients treated with sunitinib, and hypertension (5.0%), hand-foot syndrome, diarrhea, abdominal pain, skin rash, and dyspnea (3.3% each) in patients treated with sorafenib.

**Table 2:** Adverse Events (≥ 5%) among Patients Receiving First-Line Multikinase Inhibitor Treatment

	Sunitinib		Sorafenib	
	(N = 85)		(N = 60)	
**N (%) with adverse event**	**All grades**	**Grades 3/4**	**All grades**	**Grades 3/4**

Any adverse event	83 (97.6)	23 (27.1)	42 (70.0)	19 (31.7)

Fatigue/Asthenia	69 (81.2)	8 (9.4)	26 (43.3)	6 (10.0)

Mucositis/Stomatitis	50 (58.8)	2 (2.4)	16 (26.7)	0 (0.0)

Hand-foot syndrome	29 (34.1)	2 (2.4)	23 (38.3)	2 (3.3)

Diarrhea	26 (30.6)	0 (0.0)	19 (31.7)	2 (3.3)

Hypertension	35 (41.2)	3 (3.5)	6 (10.0)	3 (5.0)

Decreased taste sensation	36 (42.4)	0 (0.0)	1 (1.7)	0 (0.0)

Abdominal pain	19 (22.4)	3 (3.5)	10 (16.7)	2 (3.3)

Nausea	25 (29.4)	3 (3.5)	2 (3.3)	0 (0.0)

Lack of appetite	12 (14.1)	0 (0.0)	5 (8.3)	1 (1.7)

Pain	13 (15.3)	0 (0.0)	4 (6.7)	1 (1.7)

Anorexia	15 (17.6)	5 (5.9)	1 (1.7)	0 (0.0)

Vomiting	15 (17.6)	5 (5.9)	1 (1.7)	0 (0.0)

Hemorrhage	12 (14.1)	0 (0.0)	1 (1.7)	0 (0.0)

Constipation	6 (7.1)	0 (0.0)	6 (10.0)	1 (1.7)

Edema	12 (14.1)	1 (1.2)	0 (0.0)	0 (0.0)

Dermatitis	6 (7.1)	0 (0.0)	4 (6.7)	1 (1.7)

Anemia	5 (5.9)	1 (1.2)	3 (5.0)	1 (1.7)

Erythema	7 (8.2)	1 (1.2)	1 (1.7)	0 (0.0)

Hypothyroidism	6 (7.1)	1 (1.2)	2 (3.3)	0 (0.0)

Skin rash	2 (2.4)	0 (0.0)	6 (10.0)	2 (3.3)

Dyspnea	4 (4.7)	0 (0.0)	3 (5.0)	2 (3.3)

Hemorrhoids	5 (5.9)	0 (0.0)	2 (3.3)	0 (0.0)

Alopecia	1 (1.2)	0 (0.0)	4 (6.7)	0 (0.0)

Note: Adverse events experienced by at least 5% of patients in at least one treatment group are reported.

### Treatment Patterns

Table 3 summarizes the treatment patterns for first-line MKIs and reasons for treatment modifications. The median duration of first-line MKI treatment was 6.6 months (95% CI: 5.3, 11.1) for sunitinib and 5.8 months (95% CI: 4.1, 8.1) for sorafenib.

**Table 3:** First-line Multikinase Inhibitor Treatment Patterns and Second-line Multikinase Inhibitor Treatment

	Sunitinib	Sorafenib
	(N = 85)	(N = 60)
**Duration of treatment, months**^**1**^		

Median (95% CI)	6.6 (5.3, 11.1)	5.8 (4.1, 8.1)

Mean (SE)	9.9 (0.9)	8.3 (0.9)

**Patients who discontinued first-line treatment, N (%)**	66 (77.6)	51 (85.0)

**Reasons for discontinuation, N (%)**^**2**^		

Adverse events	10 (11.8)	3 (5.0)

Progressive disease	53 (62.4)	35 (58.3)

Other	9 (10.6)	14 (23.3)

**Patients with first-line treatment interruption, N (%)**	29 (34.1)	16 (26.7)

**Reasons for interruptions, N (%)**^**3**^		

Adverse Events	20 (23.5)	14 (23.3)

Other	15 (17.6)	3 (5.0)

**Patients with first-line dose reduction, N (%)**	30 (35.3)	23 (38.3)

**Reasons for dose reduction, N (%)**^**4**^		

Adverse Events	26 (30.6)	22 (36.7)

General clinical conditions worsening	5 (5.9)	1 (1.7)

**Patients who received second-line angiogenesis inhibitor, N (%)**	15 (17.6)	23 (38.3)

Sunitinib, N (%)	--	21 (35.0)

Sorafenib, N (%)	15 (17.6)	--

Temsirolimus, N (%)	0 (0.0)	2 (3.3)

Notes:

1. 11 patients who received sunitinib and 6 patients who received sorafenib and who had not ended first-line treatment by the time of data collection were treated as censored observations as of the date of last follow-up.

2. Patients may have more than one reason for discontinuation of first-line treatment.

3. For patients with more than one treatment interruption during the first-line treatment, the reasons for each treatment interruption are included.

4. For patients with more than one dose reduction during the first-line treatment, the reasons for each dose reduction are included.

Among patients receiving sunitinib, 77.6% discontinued treatment, 34.1% had a treatment interruption, and 35.3% had a dose reduction. In patients receiving sorafenib, 85.0% discontinued treatment, 26.7% had a treatment interruption, and 38.3% had a dose reduction. Progressive disease was the most frequently reported reason for treatment discontinuation in both groups (62.4% for sunitinib and 58.3% for sorafenib), followed by adverse events (11.8% of patients receiving sunitinib and 5% of patients receiving sorafenib). For both MKIs, adverse events were the most frequently reported reasons for treatment interruptions (23.5% for sunitinib and 23.3% for sorafenib) and dose reductions (30.6% for sunitinib and 36.7% for sorafenib).

Table 3 also describes the reasons for changes in treatment from one MKI to another. Among patients who received sunitinib as first-line MKI, 17.6% switched to sorafenib as a second-line treatment. Among patients who received sorafenib as first-line MKI treatment, 35.0% switched to second-line treatment with sunitinib and 3.3% switched to second-line treatment with temsirolimus.

Table 4 shows the adverse events reported as reasons for treatment modifications. Forty percent of patients receiving sunitinib and 45% of patients receiving sorafenib had at least one treatment modification due to adverse events. The adverse events most frequently reported as reasons for treatment discontinuation were anorexia (4.7%) and fatigue/asthenia (3.5%) in patients receiving sunitinib and fatigue/asthenia (1.7%), dyspnea (1.7%), abdominal pain (1.7%), and skin rash (1.7%) in patients receiving sorafenib. The most common adverse events reported as reasons for treatment interruption were fatigue/asthenia (9.4%), vomiting (5.9%), and diarrhea (5.9%) in patients receiving sunitinib, and diarrhea (6.7%), hand-foot syndrome (5.0%), and skin rash (5.0%) in patients receiving sorafenib. The adverse events most frequently reported as reasons for dose reductions were fatigue/asthenia (16.5%) and stomatitis/mucositis (4.7%) in patients receiving sunitinib, and fatigue/asthenia (20.0%) and hand-foot syndrome (18.3%) in patients receiving sorafenib.

**Table 4:** Adverse Events Reported as Reasons for Treatment Modifications in First-line Multikinase Inhibitor Treatment

	Sunitinib	Sorafenib
	(N = 85)	(N = 60)
**At least one treatment modification due to AEs, n (%)**	34 (40.0)	27 (45.0)

**Patients who discontinued first-line treatment due to AEs, n (%)**	10 (11.8)	3 (5.0)

**Adverse Events**^**a**^		

Anorexia	4 (4.7)	0 (0.0)

Fatigue/asthenia	3 (3.5)	1 (1.7)

Fever	2 (2.4)	0 (0.0)

Dyspnea	1 (1.2)	1 (1.7)

Abdominal pain	1 (1.2)	1 (1.7)

Skin rash	0 (0.0)	1 (1.7)

**Patients with first-line treatment interruption due to AEs, n (%)**	20 (23.5)	14 (23.3)

**Adverse Events**^**a,b**^		

Fatigue/asthenia	8 (9.4)	2 (3.3)

Diarrhea	5 (5.9)	4 (6.7)

Hand-foot syndrome	2 (2.4)	3 (5.0)

Vomiting	5 (5.9)	0 (0.0)

Dyspnea	2 (2.4)	2 (3.3)

Anemia	2 (2.4)	1 (1.7)

Hypertension	2 (2.4)	1 (1.7)

Skin rash	0 (0.0)	3 (5.0)

Stomatitis/mucositis	3 (3.5)	0 (0.0)

Gastritis	2 (2.4)	0 (0.0)

Nausea	2 (2.4)	0 (0.0)

Edema	2 (2.4)	0 (0.0)

Ulcer	2 (2.4)	0 (0.0)

**Patients with first-line dose reduction due to AEs, n (%)**	26 (30.6)	22 (36.7)

**Adverse Events**^**a,b**^		

Fatigue/asthenia	14 (16.5)	12 (20.0)

Hand-foot syndrome	3 (3.5)	11 (18.3)

Diarrhea	3 (3.5)	7 (11.7)

Stomatitis/mucositis	4 (4.7)	2 (3.3)

Vomiting	3 (3.5)	1 (1.7)

Dyspnea	1 (1.2)	2 (3.3)

Hypertension	1 (1.2)	2 (3.3)

Nausea	3 (3.5)	0 (0.0)

Pain	0 (0.0)	2 (3.3)

Stomach ache	1 (1.2)	2 (3.3)

Hiccup	2 (2.4)	0 (0.0)

Lack of appetite	0 (0.0)	2 (3.3)

Edema	2 (2.4)	0 (0.0)

Ulcer	2 (2.4)	0 (0.0)

AE = adverse event

a. Patients may have reported more than 1 adverse event leading to a treatment modification.

b. Adverse events reported as reasons for at least 2% of patients in at least 1 treatment group are reported.

## Discussion

Few published studies on MKIs have examined the safety and treatment patterns of these agents outside of clinical trial or EAP settings. This study examined the safety profiles of sunitinib and sorafenib and their association with treatment patterns as observed in a real-world clinical setting in Italy. Due to the small sample size in each treatment group and the observational nature of this study, statistical comparisons between groups are not likely to be meaningful. Hence, the results presented in this study are descriptive in nature.

It may be of interest to summarize the results from the present study in the context of safety data of these agents from either clinical trials or EAPs. However, there are differences between the population in the present study and the populations in these other studies that should be considered. For example, the present study includes patients who are MKI-naïve, who may or may not be cytokine naïve, while the clinical trial for sunitinib included only patients who were cytokine-naïve; the clinical trial for sorafenib included both cytokine-naïve and cytokine-pretreated patients [1,2]. In this way, the population in the present study may be more comparable in composition to the EAP studies where more than half of patients were cytokine-pretreated [6,11]. However, there are differences that still remain between this study and the EAPs studies. For example, the present study had a considerably higher proportion of cytokine pretreated patients in the sunitinib group than did the sunitinib EAP (80% versus 68%) [6]. With limited sample sizes, it is not possible to separate the patients in the present study by cytokine pre-treatment status. Furthermore, the proportion of patients with specific types of metastatic sites varied between studies; for example, in the current study, 16.5% of sunitinib patients had brain metastasis whereas only 7% of patients in the sunitinib EAP did [6].

One should consider that patients in the present study were referred to the center mainly for enrollment into clinical trials, and this is the priority. Since the vast majority of the patients reported here were excluded from clinical trials for several reasons (including comorbidities, not considered for the purposes of this paper), they could be considered to be a poorer prognostic group of RCC patients, definitely more close to patients in everyday clinical practice.

For any grade adverse events, the present study found fatigue/asthenia, followed by mucositis/stomatatis and decreased taste sensation as the most frequent adverse events associated with sunitinib while fatigue/asthenia, hand-foot syndrome, and diarrhea were the most frequent adverse events associated with sorafenib. This is generally consistent with the findings for the EAPs for each of these agents, where these adverse events were among the most commonly reported adverse events.

The rates for some adverse events observed in the present study were higher than may be expected compared with findings from EAPs. For example, the observed rates (any grade) for fatigue/asthenia of 81.2% and for mucositis/stomatitis of 58.8% for sunitinib appeared to be considerably higher than what may be expected based on the sunitinib EAP (37% and 28%, respectively). This finding may be due to various underlying population differences noted above (specifically, a higher rate of cytokine refractory patients in the present study). Additionally, in the present study the rates (any grade) for fatigue/asthenia of 43.3% and for hand-foot syndrome of 38.3% for sorafenib appeared to be considerably higher than the corresponding rates reported in the sorafenib EAP (grade 2+, 11% and 18.1%, respectively).

In addition, the length of time of patient follow-up and frequency of patient visits may differ between settings. Patients in clinical trials are observed for a finite period of time while patients in a naturalistic setting may have a longer observation period, which may result in more adverse events being observed in clinical settings. On the other hand, clinical trials have more vigilant surveillance that could lead to higher observed rates of adverse events compared to clinical settings, where physicians may not record all adverse events or proactively inquire patients about their adverse event experiences.

Moreover, this study examined reasons for treatment modifications. The rate of treatment discontinuation was high in both groups, with most discontinuations due to disease progression, and some discontinuations due to adverse events. Adverse events were the most frequently reported reasons for dose reductions and treatment interruptions. These results suggested that adverse events play an important role in decisions for treatment modifications.

Although fatigue/asthenia, diarrhea, hand-foot syndrome, and vomiting appeared to be the most common adverse events leading to treatment modifications, a clear pattern associated with specific adverse events and their impact on clinical decisions for treatment modification remain to be further studied.

Due to the aforementioned differences between the population of this study and that of the EAPs, and the fact that this study focused on safety and treatment patterns, the survival outcomes could not be formally compared. Furthermore, it is difficult to compare these outcomes across studies as clinical assessments to determine disease progression are conducted at different frequencies in clinical trials and observational settings; infrequent assessment for disease progression could lead to overestimating PFS. In this study, the median PFS for sunitinib was 7.3 months (95% CI 5.4-11.1), and the median OS was 14.5 months (95%CI 12.5-20.0). In the EAP, the median PFS for sunitinib was 10.9 months (95% CI 10.3-11.2), and the median OS was 18.4 months (95% CI 17.4-19.2). Median duration of sunitinib therapy in this study was 6.6 months while median follow-up duration in the EAP was 11.6 months. For patients receiving sorafenib in this study, the median PFS was 7.3 months (95% CI 5.1-11.1), and the median OS was 15.0 months (95% CI 9.5-NR). In the EAP, the median PFS was 5.5 months (95% CI 5.1-5.8), and the median OS was 11.5 months (95%CI 10.6-12.0). Median treatment duration for sorafenib in this study was 5.8 months while in the EAP it was 2.8 months. Based on these qualitative observations, the survival outcomes appear to be generally consistent with those from the EAPs.

This study has several limitations. First, the study sample was relatively small and came from a single tertiary oncology center in Italy, limiting generalizability of the study results. Future studies are needed to perform similar evaluations in patients with mRCC in other countries. Finally, because this is an observational study, the lack of randomization and the potential resulting selection bias limit the study's ability to compare between agents.

## Conclusions

In this study, patients receiving sunitinib or sorafenib frequently experienced treatment-related adverse events. Progressive disease was the most common reason for first-line MKI discontinuation, while adverse events were the most common reasons for treatment interruptions and dose reductions. While the rates of specific adverse events were different in the current study compared with the EAPs, the results from this retrospective study in a real-world observational setting corroborate findings from the EAPs that adverse events are commonly associated with sunitinib and sorafenib treatment. Together, these results may suggest a need for additional effective and more tolerable treatments for mRCC.

## List of Abbreviations

MKI: multikinase inhibitor; VEGF: vascular endothelial growth factor; U.S.: United States; PFS: progression-free survival; mRCC: metastatic renal cell carcinoma; ECOG: Eastern Cooperative Oncology Group; PS score: performance status score; EAP: expanded access program; CTCAE: National Cancer Institute's Common Terminology Criteria for Adverse Events

## Competing interests

*Financial competing interests*: Funding for this research was provided by GlaxoSmithKline, Collegeville, Pennsylvania. CP, CP, and II are employees of the IRCCS San Matteo University Hospital Foundation in Pavia, which has received research funds from Analysis Group, Inc. CC is a post-graduate student at the hospital. KC and MSD are employees of Analysis Group, Inc., which has received research grants from GlaxoSmithKline. MN is an employee of GlaxoSmithKline.

## Authors' contributions

MSD and MN contributed to the study concepts. MSD, KC, CP, II, and CC contributed to the study design. CP, CP, II, and CC contributed to data acquisition. KC contributed to quality control of data and algorithms. MSD, KC, MN, CP, CP, II, and CC contributed to data analysis and interpretation. MSD and KC contributed to statistical analysis. KC contributed to manuscript preparation. KC, MN, CP, CP, II, and CC contributed to manuscript editing. MSD, MN, CP, CP, II, and CC contributed to manuscript review.

All authors have read and approved the final manuscript.

## Pre-publication history

The pre-publication history for this paper can be accessed here:

http://www.biomedcentral.com/1471-2407/11/105/prepub
